# A prospective, multi centre, randomized clinical study to compare the efficacy and safety of Ertapenem 3 days versus Ampicillin - Sulbactam 3 days in the treatment of localized community acquired intra-abdominal infection. (T.E.A. Study: Three days Ertapenem vs three days Ampicillin-sulbactam)

**DOI:** 10.1186/1471-230X-11-42

**Published:** 2011-04-18

**Authors:** Federico Coccolini, Fausto Catena, Luca Ansaloni, Giorgio Ercolani, Salomone Di Saverio, Filippo Gazzotti, Daniel Lazzareschi, Antonio D Pinna

**Affiliations:** 1 Unit of General, Emergency and Transplant Surgery, St. Orsola-Malpighi University Hospital, Bologna, Italy; 2 Unit of General Surgery, Ospedali Riuniti, Bergamo, Italy; 3 Unit of Trauma and Emergency Surgery, Maggiore Hospital, Bologna, Italy; 4 Department of Integrative Biology, University of California, Berkeley, USA

## Abstract

**Background:**

The recommendations outlined in the latest guidelines published by the Surgical Infection Society (SIS) and the Infectious Disease Society of America (IDSA) regarding the proper duration of antibiotic therapy in patients with intra-abdominal infections are limited and non-specific. This ambiguity is due mainly to the lack of clinical trials on the topic of optimal duration of therapy. It is well known that the overuse of antibiotics has several important consequences such as increased treatment costs, reduced clinical efficacy, and above all, the increased emergence of antibiotic-resistant pathogens. Ampicillin-Sulbactam is a commonly used "first line" antibiotic for intra-abdominal infections. Ertapenem and Ampicillin-sulbactam are recommended as primary treatment agents for localized peritonitis by both the SIS and IDSA guidelines.

**Methods/Design:**

This study is a prospective multi-center randomized investigation. The study will be performed in the Departments of General, Emergency, and Transplant Surgery of Sant'Orsola-Malpighi University Hospital in Bologna, Italy, in the General Surgery Department of the Ospedali Riuniti of Bergamo, Italy, and in the Trauma and Emergency Surgery Department of Maggiore Hospital in Bologna, Italy, and will be conducted by all surgeons willing to participate in the study. The inclusion period of the study will take approximately two years before the planned number of 142 enrolled patients is reached.

**Discussion:**

Ertapenem and Ampicillin-sulbactam are recommended both as primary treatment agents for localized peritonitis by both the SIS and IDSA guidelines. As one of the discussed topic is the optimal duration of the antibiotic therapy and this ambiguity is due mainly to the lack of clinical trials on the topic, the present study aims for obtain precise data.

**Trial Registration:**

ClinicalTrials.gov: NCT00630513

## Background

The recommendations outlined in the latest guidelines published by the Surgical Infection Society (SIS) and the Infectious Disease Society of America (IDSA) regarding the proper duration of antibiotic therapy in patients with intra-abdominal infections are limited and non-specific According to these guidelines, an "antimicrobial therapy for established infections should be continued until resolution of clinical signs of infection occurs, including normalization of temperature and WBC count and return of gastrointestinal function" [1].

Such ambiguity is primarily attributable to the lack of clinical trials conducted on the topic of the optimal duration of antibiotic therapy.

These vague guidelines have manifested themselves in clinical practice; in most trials for antibiotic therapy, a fixed period ranging from 5 to 14 days is used for all patients presenting with community-acquired intra-abdominal infection, irrespective of severity of the peritonitis.

It should be noted that secondary peritonitis encompasses several different diseases and, as such, its severity has been known vary significantly [2].

It has been demonstrated that most patients enrolled in trials involving antibiotic treatment of IAI present with a mild form of the acute illness, which in 35-55% (and, in some studies, up to 70%) of cases is represented by acute appendicitis [3]. It should also be noted that many patients do not suffer from a fully developed and widely spread infection but rather experience a localized infection or simple contamination [4].

This means that many patients receive superfluous and unnecessary antibiotic treatments. Many non-random trials demonstrated that, by tailoring the duration of the antibiotic therapy to the extent of the infection as assessed during surgery, the same clinical results could be obtained in all groups of treated patients without the use of excessive antibiotic treatment regimens [5]. A recent systematic meta-analysis of twenty-eight studies on the duration of antibiotic therapy in cases of advanced pediatric appendicitis demonstrated that limiting the duration of antibiotic use to only 3 days was not associated with higher rates of intra-abdominal abscesses or wound infection [5].

In 2006, Basoli et al. demonstrated in a randomized study that a shorter duration of Ertapenem is as effective as a standard 5-day treatment regimen in patients presenting with mild-moderate peritonitis, thereby resulting in a significant reduction in the excessive use of antibiotics. These results represent a much-needed reassessment of antibiotic treatment and a conservative shift in antibiotic regimen methodology [6].

It is well known that the overuse of antibiotics has several important consequences such as increased treatment costs, reduced clinical efficacy, and above all, the increased emergence of antibiotic-resistant pathogens. The use of excessive or otherwise inappropriate antibiotic regimens may facilitate the emergence of resistant bacterial strains [7].

The fact that Ertapenem appears to be an effective monotherapy for community-acquired IAI is particularly important.

Ertapenem is a long-term acting parenteral Group I carbapenem, which is active in vitro against most aerobic and anaerobic bacteria generally associated with community-acquired infections[8,9]. Ertapenem is not active against most *Pseudomonas aeruginosa *or enterococci, but prevention of such strains is not routinely required for successful treatment of intra-abdominal infections[10,11].

In an earlier double-blind randomized clinical trial, Ertapenem proved to be just as effective as piperacillin-tazobactam [12].

Ampicillin-sulbactam (AS) is a widely used "first line" antibiotic for treating IAI.

Ertapenem and AS are recommended as single agents for localized peritonitis in both the SIS and IDSA guidelines.

The AS daily dosage is 3 g × 3 compared to Ertapenem, which is a 1 g single administration.

In Italy, 1 g of Ertapenem costs 36 euros while 1 g of AS costs only 0.23 euros.

*Escherichia coli *is the most common bacterium involved in localized community-acquired IAI; unpublished data from a retrospective analysis of 300 cases from Sant'Orsola-Malpighi's General Surgery Department reported a growing rate of resistance of *Escherichia coli *to AS as high as 30% [13].

The aim of this study is to compare the results of Ertapenem administered in a short 3-day regimen (in terms of efficacy and safety) to a short 3-day regimen of AS for patients presenting with community-acquired IAI of mild to moderate severity.

## Methods

### Design

This study is a prospective multi-center randomized investigation. The study will be performed in the Departments of General, Emergency, and Transplant Surgery of Sant'Orsola-Malpighi University Hospital of Bologna, in the General Surgery Department of the Ospedali Riuniti of Bergamo, and in the Trauma and Emergency Surgery Department of Maggiore Hospital of Bologna, and will be conducted by all surgeons willing to participate in the study. The study has been designed and will be conducted in compliance with the regulations outlined in "Good Clinical Practice" methodology.

The efficacy of a 3-day treatment regimen of Ampicillin-Sulbactam (AS 3 g × 3/day administered intravenously) is compared to a 3-day regimen of Ertapenem (1 g/day administered intravenously) for treating patients with localized peritonitis in a blinded evaluation of established efficacy-based endpoints. Evaluation of success or failure is blinded by the use of designated third party administrators who are unaware of the treatments assigned to the patients.

### Randomization

The randomization will be achieved by means of a computer-generated assignment system (a randomization algorithm). The results of this randomization will be sealed in numbered envelopes. Following the diagnosis of localized peritonitis, if the patient meets the inclusion criteria and if he/she is willing to participate in the study, the patient will sign a document verifying informed consent at which time the attending surgeon will disclose the contents sealed within the assigned envelope to determine the patient's randomly assigned treatment group.

The attending surgeon will then record the patient name and envelope number.

### Statistics

Each group will consist of 71 patients (142 patients for the entire study). All randomized patients (Intention to Treat Population) will be included in the analysis. Sample size has been calculated to reach a confidence level of 95% with a power of 80%. A sample size of 71 patients has been calculated assuming that Ertapenem can decrease the rate of "failure" from 30% to 10% as compared to AS.

The statistical analysis will be carried out using Epi Info 2000, Version 1.1 software package (Dean AG, Arner TG, Sangam S, Sunki GG, Friedman R, Lantinga M, Zubieta JC, Sullivan KM, Smith DC. Epi Info 2000, a database and statistics program for public health professionals for use with Windows 95, 98, NT, and 2000 computers; Centers for Disease Control and Prevention, Atlanta, Georgia, USA, 2000). The data generated in this study will be analyzed in two ways. Researchers will use the continuous numerical data to perform an analysis of variance (ANOVA), given that this method can discriminate between two continuous populations. Discrete data will be analyzed using the Chi-squared test or Fisher exact test, as appropriate. Differences between the two study groups will be considered statistically significant for p-values less than 0.05 (p < 0.05).

### Inclusion and Exclusion Criteria

#### Inclusion criteria

• Adult patients (>18 years of age)

• Patients requiring surgical intervention within 24 hours of diagnosis.

• Localized IAI (i.e., infection extending beyond the organ wall but confined by the closest hollow viscera, mild to moderate in severity):

• Acute appendicitis: Ruptured or perforated with abscess(es)

• Acute diverticulitis with perforation and/or abscess(es)

• Acute cholecystitis (including gangrenous cases) with either rupture or perforation

• Acute gastric and duodenal perforation (> 24 hours)

• Traumatic perforation of the intestines (> 12 hours)

• Secondary peritonitis due to perforated viscera

• Intra-abdominal abscess(es) (including those affecting both liver and spleen)

#### Exclusion criteria

• Traumatic bowel perforation requiring surgery within 12 hours

• Perforation of gastroduodenal ulcers requiring surgery within 24 hours

• Other intra-abdominal diseases in which the primary etiology was unlikely to be infectious.

• Patients lactating or pregnant

• Patients with history of allergy, hypersensitivity, or any severe reaction to the study antibiotics

• Patients with rapidly progressive or terminal illnesses;

• Patients with a history or presence of severe hepatic or renal disease (e.g. creatinine clearance < 0.5 ml/min/1.73 m2);

• Patients with a concomitant infection that would interfere with the evaluation of responses to the study antibiotics.

### Surgical Intervention

Upon patient enrollment, the severity of the disease is evaluated by means of an APACHE II score and MPI prior to the operation. Diagnosis is based on the patient's clinical symptoms and intra-operative findings, including intra-operative cultures. The drug regimen being studied begins prior to surgery. The patients undergo surgery and then receive a 3-day treatment of either Ertapenem (1 g per day) or AS (3 g, 3× per day), depending on the assigned treatment group. Administration of the antibiotic therapy is then discontinued for those patients demonstrating an improved temperature (< 37.8°C), decreased WBC (returning to the normal range), and the presence of abdominal sounds by the third day of treatment.

In order to balance the treatment groups, the patients are stratified according to the site of infection; cases are either categorized under "complicated appendicitis" or they are filed under the all-encompassing group, "all other diagnoses." To limit the proportion of cases with complicated appendicitis, we have limited the enrollment into a stratum when almost 50% of cases are enrolled. Considering that an adequate surgical source control is a crucial factor determining the outcome of this disease, a blinded panel of three surgeons is asked to impartially assess the efficacy of the surgical procedure.

Aerobic and anaerobic cultures of intra-operatively retrieved specimens are obtained at baseline and processed in the clinical microbiology laboratories of the participating hospitals. All microorganisms are isolated, cultured, and tested for in vitro susceptibility to the study antibiotic by disk diffusion or microtiter dilution. Routine susceptibility testing of strict anaerobes is not required by treatment protocols.

### Data Collection

Patient data sheets are generated containing demographic figures as well as preoperative, operative, and postoperative information.

Upon enrollment, all patients undergo physical examinations and laboratory tests, (including a CBC with a WBC, differential analysis, platelet count, serum glucose assessment, BUN, and serum creatinine test). The same procedure is performed on the third day of the treatment regimen, at the end of the treatment cycle following the resolution of pathology, and during the post-treatment follow-up analysis (or more frequently as clinically relevant). Liver function studies, serum electrolytes, and urinalysis are performed upon the discontinuation of the intravenously administered antibiotic as well as periodically throughout the treatment process as clinically indicated. When clinically indicated during the course of antibiotic therapy, blood, urine and tissue specimens from clinically relevant intra-abdominal sites are obtained for culture and susceptibility testing. Such cultures are also performed at the end of antibiotic therapy, unless there is no material available for cultures and/or no clinical evidence of infection.

The clinical outcomes of eligible patients are classified in three groups: successful (no signs or symptoms of infection and no further need for antimicrobial therapy); failure (indicating no improvement, infection progression, or death due to infection); or late failure (indicating recurrence between cessation of antibiotic therapy and follow-up). Microbiological reports are recorded for each baseline pathogen. Favorable microbiological responses involve eradication of the pathogen(s), assessed on either a documented or presumptive basis (the latter occurring when there is no material available for culture in clinically recovered patients). By contrast, unfavorable microbiologic responses involve a chronic persistence of the pathogen(s), either on a documented or presumptive basis.

Any and all adverse events (AEs) that occur during the course of the study, beginning from the time of randomization, are carefully documented. These AEs are identified and described by the primary investigators.

### Informed Consent or Information Sheet

In the informed consent document, the patient will review all relevant information about the study protocol and the confidentiality of personal data. After signing the informed consent form, the patient will fill out a questionnaire. The patients will experience minimal bureaucratic inconveniences during the enrollment procedure. No incentives are planned for the patients regarding the surgical operation or the follow-up analysis. All of the patients' medical information will remain confidential and will only be accessed by the research scientists conducting the study. The patients are permitted to withdraw from the study at any time without any further commitments or obligations.

### Ethical Approval

The study has been approved by the Ethics Committee of St. Orsola-Malpighi Hospital

### Primary Endpoint

The study's primary endpoint is to compare the failure rates of shortened Ertapenem and AS antibiotic therapies in treating localized IAI.

### Secondary Endpoints

The study's secondary endpoints include the observation and recording of onsets of any other complications as assessed intra-operatively, postoperatively, at the time of discharge, and during 7-day, 1-month, and 6-month follow-up analyses.

The late failure rates and total costs of the antibiotic therapy regimens will also be recorded.

No incentives are planned for the patients regarding the surgical operation or the follow-up analysis.

It has been estimated that the study's inclusion period will take approximately two years. According to the number of localized IAI cases reported on a monthly basis by the surgeons of Sant'Orsola-Malpighi's General Surgery Department, the inclusion period of the study will require approximately two years before the planned number of 142 enrolled patients is reached.

Interim reports will be conducted after each follow-up period.

## List of abbreviations

WBC: White Blood cell Count; IAI: Intra-Abdominal Infection; AS: Ampicilli-Sulbactam; SIS: Surgical Infection Society; IDSA: Infectious Diseases Society of America; BUN: Blood Urea Nitrogen; CBC: Complete Blood Count; MPI: Manheim Peritonitis Index; AE: Adverse Event; ANOVA: Analysis of Variance; APACHE II: Acute Physiology and Chronic Health Evaluation II

## Competing interests

The authors declare that they have no competing interests.

## Authors' contributions

CF and CF contributed equally to this work in study conception and design and in manuscript conception and draft. AL, EG, and PAD participated in the study conception and design, made critical revisions of the manuscript, contributed important scientific insight, and ultimately gave final approval. DSS and LD critically revised the manuscript and language style while DSS and GF contributed to multiple manuscript drafts, ultimately giving final approval.

All authors have read and approved the final manuscript.

## Pre-publication history

The pre-publication history for this paper can be accessed here:

http://www.biomedcentral.com/1471-230X/11/42/prepub
